# Prospects in the Application of Photodynamic Therapy in Oral Cancer and Premalignant Lesions

**DOI:** 10.3390/cancers8090083

**Published:** 2016-09-02

**Authors:** Rajan Saini, Nathan V. Lee, Kelly Y. P. Liu, Catherine F. Poh

**Affiliations:** 1 Department of Oral Biological and Medical Sciences, Faculty of Dentistry, University of British Columbia, Vancouver, BC V6T 1Z3, Canada; rsaini@dentistry.ubc.ca (R.S.); nathan.lee@alumni.ubc.ca (N.V.L.); keliu@bccrc.ca (K.Y.P.L.); 2 Department of Integrative Oncology, British Columbia Cancer Research Centre, Vancouver, BC V5Z 1L3, Canada

**Keywords:** photodynamic therapy, tumor vaccine, precancer, topical treatment, oral cancers

## Abstract

Oral cancer is a global health burden with significantly poor survival, especially when the diagnosis is at its late stage. Despite advances in current treatment modalities, there has been minimal improvement in survival rates over the last five decades. The development of local recurrence, regional failure, and the formation of second primary tumors accounts for this poor outcome. For survivors, cosmetic and functional compromises resulting from treatment are often devastating. These statistics underscore the need for novel approaches in the management of this deadly disease. Photodynamic therapy (PDT) is a treatment modality that involves administration of a light-sensitive drug, known as a photosensitizer, followed by light irradiation of an appropriate wavelength that corresponds to an absorbance band of the sensitizer. In the presence of tissue oxygen, cytotoxic free radicals that are produced cause direct tumor cell death, damage to the microvasculature, and induction of inflammatory reactions at the target sites. PDT offers a prospective new approach in controlling this disease at its various stages either as a stand-alone therapy for early lesions or as an adjuvant therapy for advanced cases. In this review, we aim to explore the applications of PDT in oral cancer therapy and to present an overview of the recent advances in PDT that can potentially reposition its utility for oral cancer treatment.

## 1. Introduction

Each year, oral cancer accounts for 300,400 new cases and 145,300 cancer related deaths [1]. Despite oral lesions occurring at easily accessible sites, diagnosis is often made at late stages, with an average 5-year survival rate below 60%. Even with current treatments, namely surgery and radiotherapy, recurrence rates for oral cancer have not improved, ranging from 30% to 47% for oral squamous cell carcinoma (SCC) [2,3]. The quality of life is also compromised after conventional oral cancer treatment, with psychosocial impacts and functional disabilities such as post-treatment tissue morbidity, xerostomia, mucositis, and fibrosis being prevalent [4,5,6,7,8]. Alternative treatment for better management of this disease is needed.

Photodynamic therapy (PDT) has been used in the treatment of cancers due to its specificity and sensitivity for tumor cells. The antitumor effects of PDT may result directly from tumor cell death, or indirectly from damage to tumor vasculature and activation of non-specific and specific immune responses against the tumor cells [9]. Due to its location and direct visibility, oral precancers and cancerous lesions are an ideal model for conventional PDT. In this review, we aim to explore the applications of PDT in early and advanced oral cancer therapy and present an overview of the recent advances in PDT that can potentially reposition the technique for its use in oral cancer therapy.

## 2. Current Applications of PDT in Oral Cancers and Precancers

With the advent of endoscopic fiber optic devices, PDT has been successfully used in the treatment of various cancers arising from the head and neck regions. Premalignant or dysplastic lesions affecting the oral mucosa are ideal candidates for PDT as large diseased areas can be treated superficially with minimal morbidity. Effects of PDT, in such cases, are found to be limited to the superficial epithelial layers while sparing the underlying connective tissue and muscle. The intact subepithelial collagen and elastin, which are needed for regeneration, promote healing with minimal scarring and excellent functional and cosmetic results [10].

### 2.1. PDT as a Topical Therapy 

We have previously reviewed the application of topical PDT, a minimally invasive and minimally toxic technique [9]. While PDT has shown promise in managing oral premalignant lesions (OPLs), the efficacy of PDT to OPLs remains elusive [5]. This is mainly due to a lack of consensus in proposed treatment protocols of PDT studies, including the light source, photosensitizer (PS), and treatment dosage and frequency. Moreover, the histology, the size, and the anatomical site of the lesions were usually not clearly described. Most importantly, the duration of the follow-up time was not consistently reported. With such heterogeneity in study designs, the authors reported ~30% of local recurrence rate post-treatment; continuous tobacco consumption was thought to be a risk factor for recurrence [5]. With similar limitations, a recent meta-analysis on the efficacy of PDT and surgery in early-stage oral cancer reported that there was no statistically significant difference in recurrence rates between two interventions [4]. We agree with the authors that invasive surgical procedures are often accompanied by higher morbidity and have a greater negative impact on quality of life, compared to PDT; and that to further address the efficacy of PDT, a well-designed randomized control trial is needed. We have previously reported a simple hand-held device, the VELscope^®^ (LED Dental Ltd., Vancouver, BC, Canada), uses tissue autofluorescence to detect subclinical high-risk field changes that are associated with oral cancerous and precancerous lesions [11]. With the clearly demarcated lesional extension, the use of topical PDT may provide an exciting new modality for the treatment of these early oral lesions, whereby wide fields of clinically visible as well as inconspicuous dysplastic mucosa can be treated, even repeatedly, without any cumulative toxicity.

### 2.2. PDT as a Primary Treatment: Advantages and Limitations

Many studies have reviewed the efficacy of PDT for the treatment of cancers arising from the head and neck region, including the oral cavity [7,8,12,13]. Table 1 summarizes these studies that have used PDT for the treatment of early and advanced primary oral cancers in chronological order from 1987 and with a minimum of three patients with at least 3-month follow-up. From the results of the retrospective studies and clinical trials, it can be concluded that PDT is well-suited as a primary or alternative treatment modality for early oral cancer without nodal metastasis (i.e., T1 and T2 tumors) and is associated with significantly less morbidity compared to conventional therapy. Superficial cancers that are within the permeability range of the light source (i.e., 0.5–1 cm) seem to show the best response. Advantages of PDT over conventional therapies, such as surgery, radiotherapy, and chemotherapy include minimal invasiveness, organ-sparing potential, excellent long-term functional and cosmetic results with improved quality of life, feasibility of repeating treatment at the same site for recurring lesions, minimal scarring after treatment, cost effectiveness, and simplicity of technique [14,15]. In the case of relapse or a development of new primary tumor in the area previously treated by PDT, treatment can be repeated at the same area multiple times without cumulative toxicity, in contrast to ionizing radiation or surgery where such retreatments result in extensive morbidity. In addition, the use of conventional therapies does not preclude the use of PDT and use of PDT does not compromise future surgical interventions or radiation therapy [16].

### 2.3. PDT as an Adjuvant and/or Combined Modality 

PDT has the potential to be used as an adjuvant therapy for the treatment of surgical margins following resection of T3 and T4 head and neck cancers via superficial or interstitial light application [13]. The use of PDT in conjunction with conventional therapies warrants further investigation. PDT may be used to treat primary localized lesions alongside surgery and/or radiotherapy in cases involving nodal metastasis [8]. Recent in vitro and in vivo studies have used PDT in combination with other chemopreventive agents [34,35,36,37]. These combination therapies have shown enhanced anticancer effects, likely due to the fact that a multifactorial disease such as cancer involves various pathological pathways. Therefore, a combination of treatment modalities can be used to target different disease processes, causing cell death via diverse mechanisms. Furthermore, the combination of different modalities can synergistically enhance selectivity and efficacy in comparison to either of the stand-alone therapies. This can eventually help to reduce the amount of cytotoxic drugs given to patients, resulting in reduced morbidity from side effects [37].

### 2.4. PDT as a Palliative Treatment Modality Using Interstitial PDT (iPDT)

PDT may also play an effective role in the palliative and curative treatment of advanced refractory head and neck cancers. Interstitial light delivery systems (iPDT) have been used to debulk large tumors [38,39,40]. iPDT involves delivery of light directly into solid tumors via multiple laser fibres through needles placed under image guidance [23,41]. Interstitial light delivery is suitable for the treatment of large deep tumors that are inaccessible to surgery or would require extensive surgical resection that may cause damage to adjacent vital structures. Meta-tetra (hydroxyphenyl) chlorin (mTHPC) iPDT has been used as palliative treatment in recurrent or persistent head and neck cancers that did not respond to conventional therapies [42]. In a study reported by Lou et al., out of a total of 45 patients treated by iPDT, nine achieved complete remission (CR), while symptomatic relief in the form of reduced pain and bleeding or tumor debulking was reported in 24 patients. In another study, 20 patients with recurrent base of tongue cancers were treated using iPDT. At 6-month follow-up, nine patients had shown CR [43]. These findings suggest that iPDT can be used as a curative modality in addition to palliative care for untreatable advanced head and neck cancers.

### 2.5. PDT as a Surveillance Modality to Maintain Cancer Free Status

Various in vitro and in vivo studies have demonstrated the significant effect of PDT on the development of adaptive immunity. The first study that showed memory immunity after PDT-induced immune stimulation in a mouse model was reported by Canti et al. [44]. They demonstrated that immunocompetent mice with primary tumors treated with PDT were able to survive when rechallenged with cells isolated from tumor-draining lymph nodes. Subsequent studies have clearly established the role of adaptive immunity triggered by PDT in controlling tumor recurrence. The efficacy of the treatment was found to be correlated with increased tumor infiltration with CD8^+^ T-cells after PDT [45].

Dendritic cells (DCs) are considered the most important antigen presenting cell (APC) and play a significant role in antitumor immune response by activation of CD8^+^ cytotoxic T-cells [46]. DCs activated in response to PDT travel to tumor-draining lymph nodes, where they are known to stimulate T-cell activation [47]. Saji et al. studied the combination of PDT with intratumoral injection of DCs and found synergistically enhanced tumor cure rates as compared to individual therapies. The immune response produced by combination therapy was found to confer systemic antitumor effects that could induce the regression of distant untreated tumors [48]. PDT’s surveillance function is elaborated in Section 4.5.

## 3. Uniqueness of the Oral Cavity in the Application of PDT

### 3.1. Visualization for Difficult-to-Reach Areas in the Mouth

Meticulous delivery of precise and uniform light dosage in specific regions of the mouth for prolonged and repetitive sessions can be challenging. Slight movements of the light device or the patient can cause inconsistency in the distance between the light source target tissue resulting in significant variations in fluence rate and total light dose delivered. Furthermore, delivery of light at the right angles for proper PS activation (by avoiding the formation of shadows) to mucosal surfaces of less accessible anatomical sites, such as the tonsils, posterior or base of tongue, and oropharynx, can be difficult.

Transoral robotic surgery (TORS) is a minimally invasive, FDA-approved, computer-guided mechanically precise procedure used to assist surgeries of oral and throat cancers [49,50]. Recently, TORS technology has been shown to aid PDT in the oropharynx [51]. A 5-fold increase in the light dose and fluence rate due to a decrease in light scattering in the oropharynx was observed. No adverse events were noticed after four weeks of follow-up. The authors also concluded that the use of TORS could extend the application of PDT to the oral cavity by improving visualization along with accurate and constant fluence rate light delivery in hard-to-reach areas of the mouth. In future, a “computer-guided” light delivery system attached to the TORS may improve treatment effectiveness.

### 3.2. Minimizing the Damage to Normal Adjacent Mucosa

To minimize side effects, it is imperative to prevent damage to normal mucosa during PDT. Besides the use of systemic PSs which does not restrictively localize in tumor tissues, light scattering from the primary target site can result in unplanned light delivery to the adjacent mucosa, causing unnecessary ulceration and mucositis [15]. Undulated contours of the oral cavity further aggravate the effects from light scattering. In addition to improving the specificity of PS to target tissues, using physical barriers to prevent illuminating healthy tissues and devices which focus the delivery of light to the targeted regions may decrease unwanted injury to adjacent normal tissue.

Many approaches have been employed to minimize the cytotoxic effect of PDT on non-diseased adjacent mucosa. These methods, based on the absorbing incident and reflected light, include shielding the area with black, thermal-resistant material, such as wax or a saline-soaked surgical packing which can be molded to cover the adjacent mucosal surfaces [52].

To facilitate the light delivery, various illumination devices for PDT of the oral cavity have been introduced. These include a tailored reflector, light pipe, and cylinder reflector [53,54]. These devices, with average irradiance at ~50 mW/cm^2^ and reduced spatial non-uniformities, provide more uniform and reproducible doses; thus eliminating the need to protect the entire oral cavity. More importantly, as the light source is immobilized by direct contact with the treatment field, its vulnerability to patient motion is greatly reduced. For these reasons, these devices have been proposed to be effective in treating lesions on flat surfaces such as the buccal mucosa, and dorsal and ventral surfaces of the tongue, as well as those on soft, curved surfaces, such as the lateral tongue. In the foreseeable future, the inventors of these devices predict the development of a family of light sources will be available at the clinician’s disposal that could be used to treat lesions of different sizes at various geographic locations in the oral cavity.

## 4. Recent Developments in PDT

Significant advances have been made in recent years to improve the efficacy of PDT by increasing the specificity of the PS and by formulating new light source devices capable of delivering uniform illumination. This section highlights a few recent advances in PDT that should be considered and applied to oral cancer treatment.

### 4.1. Targeted PS Delivery 

In the era of precision medicine, to increase specificity and minimize toxicity to the normal tissues, PSs are conjugated with targeting moieties. One such technique is to couple PSs to monoclonal antibodies (MAbs) that are directed against tumor-associated antigens (TAAs) specifically overexpressed in cancer cells [55]. These TAAs include oncofetal antigens [56], growth factor receptors [57], and receptors for signal transduction pathways [58]. Coupling with MAbs directly or indirectly through the use of polymers [59] can help PS to localize, accumulate, and bind specifically to targeted TAA expressing tumor cells and avoid localization into healthy cells. These targeted approaches have led to the development of third-generation PSs. Conjugating PSs to specific targeting components can also be associated with other advantages such as increased solubility in aqueous solutions, greater and faster tissue penetration, more effective clearing of the PS from circulation, and significantly lower kidney uptake [60,61]. Increasing biodistribution and localization may also increase anti-tumor efficacy.

For cancers and precancers of the head and neck, EGFR overexpression has been frequently reported [62]. Thus, targeting EGFR with photoactive molecules linked to anti-EGFR antibodies may selectively destroy cancer cells whilst sparing adjacent normal cells expressing low levels of EGFR [63]. In addition, MAb-bound fluorescent dye can also allow the monitoring of response to therapy [64]. Treatment of a mouse model of human ovarian cancer with a combination of PDT and anti-EGFR MAb C225 has been shown to cause a ten-fold reduction in mean tumor burden [65]. Additionally, a combination regimen showed synergistically enhanced survival compared to PDT or C225 alone. Hence, targeted PS delivery may help to increase its accumulation in the target tissues and substantially improve the therapy efficacy.

### 4.2. Nanotechnology in PDT

In recent years, nanoparticle (NP)-based approaches in PDT has shown promising results in improving disease outcomes. Functionally, the use of NPs in PDT is broadly divided into two classes: (1) NPs that are active participants in PS excitation, and (2) NPs that passively act as carriers for conventional PSs [66]. An example of the former is quantum dots (QDs) which are NP imaging probes composed of semiconductor materials such as cadmium selenide (CdSe) with high quantum yields, fluorescent emission properties, and low photobleaching [67]. QDs have been shown to generate singlet oxygen-causing cytotoxic effects on cells [68].

To increase the selectivity of PSs towards cancerous cells, NP can be used as drug carriers by either encapsulating them onto the surface of the PS or by covalently bounding the PS to the NP surface. With this approach, the PS can be delivered to the target site in a more selective manner with minimal toxicity and minimal damage to the normal tissues [69]. Several types of NPs have been introduced and applied in PDT such as liposomes, oil-dispersions, polymeric particles, hydrophilic polymer-PS conjugates, chitosan, silica, low-density lipoproteins, cyclodextrins, polylactic acid, and gold NPs. Besides being non-toxic and biocompatible, these NPs also have small and uniform pore size, and large pore volume with large surface areas. These properties make them suitable carriers for PS. For example, they can be used to deliver large amounts of PS or PS-MAb conjugates to the target site or to different targeting sites such as cancer cells and blood vessels. It could also be possible to give combinational therapies by combining the PS with a non-photoactive cytotoxic agent [70].

Another innovative approach of using NPs in combination therapy is a technique called NP self-lighting photodynamic therapy (NSLPDT) which combines radiation therapy with PDT [71]. This approach uses scintillation luminescent NPs which, upon exposure to ionizing radiation, emits luminescence, which in turn activates the PS and produces singlet oxygen. This recently introduced approach may provide a simple, efficient and less expensive method than irradiation or PDT stand-alone therapies. The synergistic therapeutic benefits of the NSLPDT produce more effective destruction with the use of lower doses of radiation. As ionizing radiation has deeper penetration compared to visible light, it can be used to treat deep-seated tumors. Similarly, photon-upconverting NPs (PUNPs) [72] can also be used for PDT in deep tissues as they are able to emit higher-energy photons when illuminated by lower-energy photons in a multiphoton process. [72,73].

A further possibility is to magnetically guide NP carriers to target sites [74]. An example of these carriers recently introduced is gold NPs which were used as vehicle delivering 5-ALA to increase the selectivity and efficiency of PDT [75]. Increase in the accumulation of protoporphyrin IX (PpIX) in tumor cells with a subsequent increase in cytotoxicity up to 50% was observed as compared to 5-ALA alone. In addition, the use of 5-ALA-conjugated NPs also increased the selective destruction of tumor cells.

In summary, NPs have shown promising results to address the key limitations of PDT by increasing selective accumulation into targeted tissues and penetration to deeper tissues. NPs actively participate by acting as PSs or as energy transducers by absorbing light or irradiation to excite attached PSs. This new technology holds immense potential in revolutionizing PDT and bringing it to the forefront of cancer treatment in the near future.

### 4.3. Vascular Targeted PDT

In recent years, a promising therapy that involves combination of a vascular targeted approach and PDT has been introduced [76]. Vascular targeted PDT (VTP) involves the use of PSs, such as TOOKAD^®^ (Steba Biotech, Luxembourg City, Luxembourg) and Visudyne^®^ (QLT Ophthalmics, Inc., Vancouver, BC Canada), which are selectively retained in neovasculature of targeted tumors, resulting in a preferential vascular response [76]. With a short drug-light interval (DLI) usually within 30 min of the PS’s injection [77], VTP increases the permeability of targeted blood vessels through the formation of endothelial intercellular gaps. Loss of endothelial cell barrier function following the vascular collapse and tissue hemorrhage ultimately leads to tumor destruction [78]. In comparison to conventional anti-cancer therapies directed against cancer cells, targeting tumor vasculature appears to be a more efficient approach in killing cancer cells and has a lower probability of developing drug resistance [76].

### 4.4. Two-Photon PDT

To achieve greater precision and deeper tissue penetration, new PSs capable of absorbing two photons have been developed [79,80]. In two-photon (2-γ) PDT, ultrafast pulses of near infrared light are used such that two photons of relatively low but identical energy are simultaneously absorbed by the PS. As each photon contributes to one-half of the excitation energy, longer wavelength is needed for enough energy to produce a singlet oxygen, allowing light to penetrate deeper into tissue due to lower scattering and absorption [81]. Starkey et al. reported effective PDT at depths up to 2 cm in tumor xenografts of mice [79]. Moreover, the PS activation by 2-γ PDT has been reported to be more localized than 1-γ activation, thus improving specificity [82]. The use of two-photon excitation has been utilized to target and selectively occlude blood vessels associated with neoplastic tissues while reducing the damage to adjacent normal tissues [80].

### 4.5. PDT-based Tumor-Vaccines

Cancer immunotherapy is an emerging field in oncology research that stimulates the host’s own immune system to combat the disease. Cancer vaccines are a subset immunotherapy treatment similar to a bacterial or viral vaccine, except it targets tumor cells instead. Therapeutic cancer vaccines can be divided into two classes: molecular vaccines and cellular vaccines. Unlike molecular-type cancer vaccinations that use foreign macromolecules, cellular vaccines reinfuse host tumor cells to produce a personalized tumor vaccine. PDT-based tumor cell vaccines (PDT T-vaccines) modify and devitalize tumor cells to induce the host’s own immune system to specifically respond to the tumor cells, ushering in a prospective form of personalized medicine.

#### 4.5.1. Mechanism

The immune stimulatory effects exhibited by PDT have been exploited to prepare cancer vaccines in vitro using tumor cell lysates or whole devitalized tumor cells. Patients do not undergo direct PDT treatment; instead, a part of the resected tumor tissue is exposed to PDT ex vivo and the vaccine material that is produced is administered back to the patient from which the tumor tissue originated (autologous vaccine) or to another patient (allogeneic vaccine) [83].

As a T-vaccine preparation method for tumor-matched cells, PDT was found to have better efficacy comparing to lysis, hyperthermia, freezing/thawing cycles, UV light, or x-rays. Zhang et al. demonstrated that mice given hematoporphyrin monomethyl (HMME) PDT T-vaccines had increased levels of serum CD4, CD8, and CD19 cells compared to vaccines prepared by freezing/thawing and the control group [84]. Gollnick et al. demonstrated that PDT-generated tumor cell lysates could stimulate the maturation of DCs and induce a cytotoxic T-cell response [85]. Furthermore, PDT-treated tumor cells were found to be opsonised by complement C3 that increased the efficacy of PDT-generated T-vaccine [86]. Recently, a rise in serum corticosterone levels was detected 24 hours after PDT vaccine treatment in an SCCVII mouse squamous cell carcinoma model, which showed the engagement of glucocorticoid hormones as a host immune response [87].

T-vaccines may exhibit polyvalent responses as the extracted cells can express multiple tumor antigens and release immunogenic signals after modification. This allows both innate (nonspecific) and adaptive (acquired) immune responses (that are not Major Histocompatibility Complex class I or II limited) to target the re-inoculated tumor cells as well as the primary tumor. With this, polyvalent T-vaccines can potentially avoid drug resistance resulting from tumor antigen downregulation [83].

PDT can also trigger cell surface exposure of damage-associated molecular patterns (DAMPs), molecules that are detected by the innate immune system. DAMPs, which may also be released into the tumor microenvironment, act as “danger signals” that trigger an inflammatory response, but also lead to DC activation and an adaptive immune system response. Calreticulin (CRT), high-mobility group box 1 protein (HMGB1), ceramide, sphingosine-1-phosphate (S1P), heat shock protein (Hsp) 70 and 90, and adenosine triphosphate (ATP) molecules were found to act as DAMPs after PDT exposure [88,89]. Both CRT and HMGB1 recruit macrophages and DCs, leading to phagocytosis of tumor cells and immunogenic cell death, specifically cytotoxic T-cell infiltration [90]. Ceramide and S1P expression was proportional to PDT dose. DAMPs triggering both macrophage and DC activation via nuclear factor kappa-light-chain-enhancer of activated B cells (NF-κB) is due to the photooxidative stress [89]. Hsp70 promoted opsonisation of tumor cells with complement proteins for nonspecific destruction. It was found that DC stimulation correlated with Hsp70 expression induced by T-vaccines [91]. Hsp90 expressed on the tumor cell surface or secreted into the extracellular space was found to activate DC antigen processing and presentation, inhibit TGF-β, and stimulate immunological cytokine production [92]. Other immune mediators released by PDT-based T-vaccines include TNF-α, IL-1, and IL-6. Of note, only PDT T-vaccines were able to activate DCs to express IL-12, which is vital for the cytotoxic T-cell response [85]. Therapeutic T-vaccines rely on DAMP and cytokine production leading to maturation of DCs which activate cytotoxic T-cells.

#### 4.5.2. Potential Clinical Application in Oral Cancer Treatment

PDT T-vaccines have numerous uses due to their polyvalency and specificity for the patient’s own tumor. They can serve as an adjuvant treatment after surgery, a tool for cancer surveillance, and a palliative treatment modality. For inaccessible tumors or residual diseases in large inoperable tumors, conventional radiation therapy can have objectionable side effects. When combined with surgery, PDT T-vaccines demonstrated greater survival, tumor response, and lesser recurrence in mice than surgery alone [93]. PDT T-vaccines are ideal adjuvant for surgical treatment of oral cancers as they are associated with good efficacy and less morbidity seen in radiation or surgery.

For cancer surveillance, PDT T-vaccines can be used to control the proliferation of remaining tumor cells below clinical detection. PDT T-vaccines have shown its ability to inhibit the growth of both new and established tumors; the effect is systemic and can be long-term. Korbelik et al. found that the effectiveness of inoculation at distal sites was comparable to injections proximal to the primary lesion, demonstrating a systemic cytotoxic T-cell response. Mice that were cured with a chlorin e6 (Ce6) PDT-killed cancer vaccine did not develop cancer after tumor rechallenge. This suggests cytotoxic T-cells were able to develop immune memory against the primary tumor cells and may play a role in cancer immunosurveillance [94].

#### 4.5.3. Considerations for Clinical Trials

Several key factors need to be considered prior to the application of PDT T-vaccines. First is the selection of an immunocompetent host. It was found that the T-vaccine promoted a complement system and cytotoxic T-cell response against the primary tumor; vaccine efficacy was supported by an accumulation of T-cells in regressing tumors but not in unresponsive tumors. In cytotoxic T-cell suppressed mice, the cancer vaccine had decreased effect. Furthermore, treating mice with mismatched PDT-killed tumor cells was ineffective, indicating that T-cell tumor antigen recognition played a significant role in cancer vaccination [86].

Second is the identification of interim surrogate biomarkers to assess the efficacy of the immune response to T-vaccines in vivo. One possibility is by observing delayed type hypersensitivity (DTH) reactions, which may be an indicator of systemic T-cell-mediated immunity. DTH response, measured in millimeters of skin induration, has positively correlated with overall survival [95]. T-vaccines with significant DTH responses were associated with improved survival due to the development of systemic cell-mediated immunity [96]. Changes in cytotoxic T-cell count can also be used to measure vaccine efficacy. However, using single time-point counts of circulating immune cells may not be the ideal biomarker to reflect the entire immune response. Fong et al. recommended that future studies should focus on measuring the levels of local T-cells or gene expression and circulating tumor antigens to gauge the impact of cancer vaccines [97].

Third, the choice of PS and light dose specifies the dominant form of tumor cell death, either apoptosis or necrosis. An increased concentration of PS may not necessarily have any therapeutic significance [86,98]. The PDT T-vaccine produced a greater CD4/CD8 ratio and percentage of natural killer (NK) cells in the spleen, as well as increased IFN-γ and IL-1 levels than in control mice, and even greater values with increased light dose. Overall, anti-cancer immunity was dependent on the PDT T-vaccine’s ability to enhance cytotoxic T-cell differentiation [98].

In summary, PDT has demonstrated the ability to produce polyvalent tumor lysates that have potential to upregulate tumor antigens, neoantigens, and DAMPs. Only PDT was able to trigger proper DC maturation leading to an effective cytotoxic T-cell immune response [85]. Since the photooxidative damage occurs in vitro, there is no risk of normal tissue damage. Taking ex vivo tumor cells directly from the patient means that therapeutic cancer vaccines can be personalized for each patient and targeted against specific tumor cells. All of these factors are required to overcome tumor cell immunosuppression, which is a major challenge to existing immunotherapies. Prospective studies should include clinical trials of PDT T-vaccines and efficacy measurement protocols.

## 5. Conclusions

Due to the extensive spread of lesions, multiple foci of disease, and frequent recurrence, treatment of superficial oral cancers represents a tremendous challenge. In light of the evidence available from various retrospective and prospective studies, it can be concluded that PDT offers an exciting approach for the management of early malignant lesions as a stand-alone modality or as an adjuvant therapy in combination with other therapeutic approaches. It may also improve the quality of life for inoperable, advanced, or resistant cancers. For these reasons, we may also have higher patient compliance. Together with new developments in PS delivery and T-vaccines, as well as a better integrative knowledge in cell death pathways at the molecular level, mechanisms of antivascular events, and their impact on the development of the immune response, improved therapies based on PDT-mediated tumor damage can be developed. Randomized clinical trials comparing the use of PDT to conventional treatments in oral cancer patients are needed. This will allow PDT to become one of the mainstream curative treatments of choice for early lesions as well as a palliative treatment for advanced, relapsed, or treatment-resistant head and neck cancers.

## Figures and Tables

**Table 1:** Summary of the use of photodynamic therapy in oral cancer with intent-to-cure treatment and over 3-month follow-up (in chronological order).

Year	Photosensitizer	Stage	Patients /Tumour Sites (n)	Complete Response (n, %)	Follow-up time (months)	References
1987	Photofrin II	T1/T2	8	7 (88)	7–18	[17]
1991	Photofrin	T1	23	20 (87)	8–53	[18]
1996	ALA *	SCC	6	1 (17)	76–88	[15]
1997	Foscan	T1/T2 T3/T4	13 7	6 (46) 4 (57)	6–22	[19]
2001	Photofrin	T1/T2	10	8 (80)	4–115	[20]
2003	Foscan	T1/T2	25	21 (84)	12–69	[21]
2003	Foscan	T1/T2 T3	7 2	7 (100) 1 (50)	6–48 3–12	[22]
2004	Foscan	T1/T2	85	72 (85)	12–24	[23]
2007	Foscan	T1/T2	20	12 (60)	6–105	[24]
2009	Photofrin	T1	11	10 (91)	7–52	[25]
2010	Photofrin	T1/T2	135	129 (96)	8–211	[13]
2011	Foscan	T1/T2	38	26 (68)	60	[26]
2011	Foscan	T3/T4	21	1 (5)	21–45	[27]
2011	mTHPC *	T1/T2	145	99 (68)	60	[28]
2012	mTHPC *	T1/T2 T3/T4	4 7	3 (75) 2 (29)	6–80	[29]
2013	mTHPC *	T1 T2	126 30	180 (86) 19 (63)	33 (median)	[30]
2013	Photofrin	T1/T2	18	17 (94)	24	[31]
2013	HPPH *	T1	20	17 (85)	5–40	[32]
2016	Photofrin	T1/T2 T3	29 3	28 (88) overall	68–158	[33]

* Abbreviations: ALA, Aminolevulinic acid; mTHPC: meso-tetrahydroxyphenylchlorin; HPPH: 3-(1′-hexyloxy-ethyl) pyropheophorbide; counting only those treated with light dose ≥ 100 J/cm^2^.
